# ULK1: A Promising Biomarker in Predicting Poor Prognosis and Therapeutic Response in Human Nasopharygeal Carcinoma

**DOI:** 10.1371/journal.pone.0117375

**Published:** 2015-02-25

**Authors:** Miao Yun, Hai-Yan Bai, Jia-Xing Zhang, Jian Rong, Hui-Wen Weng, Zhou-San Zheng, Yi Xu, Zhu-Ting Tong, Xiao-Xia Huang, Yi-Ji Liao, Shi-Juan Mai, Sheng Ye, Dan Xie

**Affiliations:** 1 The State Key Laboratory of Oncology in South China, Cancer Center, Sun Yat-sen University, Guangzhou, PR China; 2 Department of Oncology, the First Affiliated Hospital, Sun Yat-sen University, Guangzhou, Guangzhou, PR China; 3 Department of extracorporeal circulation, the First Affiliated Hospital, Sun Yat-sen University, Guangzhou, Guangzhou, PR China; 4 Department of Radiotherapy, the First Affiliated Hospital, Anhui Medical University, Hefei, PR China; Ospedale Pediatrico Bambino Gesu', ITALY

## Abstract

Plenty of studies have established that dysregulation of autophagy plays an essential role in cancer progression. The autophagy-related proteins have been reported to be closely associated with human cancer patients’ prognosis. We explored the expression dynamics and prognostic value of autophagy-related protein ULK1 by immunochemistry (IHC) method in two independent cohorts of nasopharygeal carcinoma (NPC) cases. The X-tile program was applied to determine the optimal cut-off value in the training cohort. This derived cutoff value was then subjected to analysis the association of ULK1 expression with patients’ clinical characteristics and survival outcome in the validation cohort and overall cases. High ULK1 expression was closely associated with aggressive clinical feature of NPC patients. Furthermore, high expression of ULK1 was observed more frequently in therapeutic resistant group than that in therapeutic effective group. Our univariate and multivariate analysis also showed that higher ULK1 expression predicted inferior disease-specific survival (DSS) (P<0.05). Consequently, a new clinicopathologic prognostic model with 3 poor prognostic factors (ie, ULK1 expression, overall clinical stage and therapeutic response) could significantly stratify risk (low, intermediate and high) for DSS in NPC patients (P<0.001). These findings provide evidence that, the examination of ULK1 expression by IHC method, could serve as an effective additional tool for predicting therapeutic response and patients’ survival outcome in NPC patients.

## Introduction

Nasopharyngeal carcinoma (NPC), an Epstein-Barr virus (EBV)-related head and neck cancer, exhibits a high prevalence in Southeastern Asia and remains one of the leading lethal malignancies in the Cantonese region of Southern China[1,2]. Compared with other head and neck cancers, the majority of NPC patients display adjacent region invasion as well as neck lymph nodes metastasis at the time of diagnosis[3]. Early-stage NPC is highly radiocurable. For locally advanced NPC, platinum-based induction chemotherapy (IC), followed by radiochemotherapy (RCT) or radiotherapy (RT) have become the backbone therapy recently, however, the survival outcome of patients with advanced stage remains poor[4,5,6]. The poor prognosis is in part related to the development of therapy resistance during conservative treatment[7,8,9]. Thus, plenty of study has focus on uncovering predictors of therapeutic response in NPC, which could identify patients who could benefit from a conservative treatment. To date, however, the promising biomarkers with great value in predicting patients’ therapy efficiency still remains substantially limited.

Autophagy is an evolutionarily conserved cellular catabolic process that is characterized by the delivery of cytosolic material and organelles to lysosomes for bulk degradation[10,11]. Dysregulation of autophagy is associated with diverse disease, including cancer, neuronal degeneration, myopathies, and the adaptive immune response to various pathogens. Intriguingly, the function of autophagy in tumorigenesis is complicated and might have opposite consequences for tumor survival depending on certain circumstances[12]. Activation of autophagy may function as a tumor suppressor by degrading defective organelles and other cellular components[13,14]. On the other hand, this pathway could also be exploited by cancer cells to generate nutrients and energy during nutrient starvation, hypoxia, or other therapeutic stress reactions, and generally protects against cell death, facilitating adaptive survival[15,16]. Products of a series of autophagy genes (ATGs) mediate and regulate various aspects of autophagy[17]. In mammals, five Atg1 homologues have been identified as uncoordinated (UNC) 51-like kinase 1 to 4 and STK36. ULK1, one of the core human autophagy-related genes, located on chromosome 12q24.3, is a serine/threonine kinase, which promote autophagy signaling[18,19,20]. Under nutrient-rich conditions, the target of rapamycin (TOR) phosphorylates both ULK1 and ATG12, which represses ULK1 kinase activity and thus lead to autophary inhibition[21,22]. On the converse, upon nutrient deprivation, ULK1 is activated by the activated AMP activated protein kinase (AMPK) and subsequently lead to initiation of autophagy[23].

Previous studies have found that elevated ULK1 expression in human cancers is associated with poor clinical outcome, such as esophageal squamous cell carcinoma (ESCC) and breast cancer[24,25]. In light of its dual function as promising prognostic biomarker as well as novel molecular target for cancer therapy, ULK1 had attracted substantial attention over the last several years. However, till now, no study has reported the expression dynamics and prognostic value of ULK1 in NPC. Thus, we took upon the present study to explore the expression pattern of ULK1 in two independent cohorts of NPC.

## Materials and Methods

### Ethics statement

The study was approved by the Institute Research Medical Ethics Committee of Sun Yan-Sun University Cancer Center (Guangzhou, China) and the First Affiliated Hospital of Anhui Medical University (Hefei, China). No informed consent (written or verbal) was obtained for use of retrospective tissue samples from the patients in the study, since most of the patients were deceased and informed consent was not deemed necessary and waived by the Ethics Committee.

### Patients and tissue specimens

Tissues were obtained from two independent NPC patents cohorts. In the training cohort, a total of 335 cases of formalin-fixed, paraffin-embedded specimens were collected from primary NPC patients between January 2001 and December 2003 at the Sun Yan-Sun University Cancer Center (Guangzhou, China). In addition, 45 samples of normal nasopharyngeal mucosa were used for controls. Ten fresh pairs of NPC tissue and the matched adjacent non-neoplastic nasopharyngeal mucosa tissue (ANT) were frozen and stored in liquid nitrogen until further use. We also collected 25 recurrent NPC and 23 liver/lung distant metastasis NPC samples with the paired primary NPC tissues. In parallel, another 215 NPC patients were selected into the validation cohort. Samples from this patient cohort were obtained from the First Affiliated Hospital of Anhui Medical University (Hefei, China) and Sun Yan-Sun University Cancer Center (Guangzhou, China) diagnosed between January 2002 and December 2006. All the cases selected were based on availability of biopsy specimens and follow-up data, no previous treatment, malignant disease or a second primary tumor; without radiotherapy, chemotherapy and surgery treatment history; Karnofsky≥70.

### Treatment

In our RT group, the radiotherapy was administered for a total dose of 66–78Gy (2 Gy/fraction, 5 days a week). The neck received 50 Gy for lymph node-negative invaded cases and 60 to 70 Gy for lymph node-positive invaded cases. In the IC/RT group, patient received two cycles of PF regimen (floxuridine 750 mg/m2, d1–5; cisplatin 35–40 mg/m2 d1–3) chemotherapy and underwent radiotherapy thereafter at 1 week interval. In IC/RCT group, one week after completion of two cycles of PF regimen, patient received radiotherapy and concurrent cisplatin (35 mg/m2 weekly) chemotherapy.

### Evaluation and follow-up

The RT or RCT response was evaluated clinically for primary lesions based on fiber optic nasopharyngeal and MRI one month after treatment according to the following criteria. Complete response (CR) was defined as the complete resolution of all assessable lesions. Partial response (PR) was defined as a reduction by 50% or more of the sum of the lesions and no progression of assessable lesions. No change (NC) was indicated by a reduction <50% or increase <25% in tumor size. All these conditions had to last for at least 4 weeks and no appearance of new lesions. Progressive disease (PD) was defined as an increase ≥25% in tumor size or the appearance of new lesions.

The patients were followed up strictly in outpatient clinics: every 3 month for the first year and then every 6 months for the next 2 years, and finally annually. The disease-specific survival (DSS) was defined as the time from diagnosis to the date of cancer-related death or when censured at the latest date if patients were still alive.

### Immunohistochemistry (IHC)

IHC analysis was performed to examine ULK1 expression level in NPC specimens. The detailed staining protocol was described previously[26]. Primary antibody against ULK1 (Abcam, #ab65056, Cambridge, UK) was applied in this study. Two independent pathologists blinded to the clinicopathological information performed the immunoreactivity score (IRS) for ULK1 expression using semiquantitative scale [27], (i) percentage of positive tumor cells in the tumor tissue: 0 (0–5%), 1 (6–25%), 2 (25–50%), 3 (51–75%) and 4 (76–100%); and (ii) signal intensity: 0 (no signal), 1 (weak), 2 (moderate) and 3 (marked). The IRS was calculated by multiplying the score for the percentage of positive cells by the intensity score (range, 0–12). The average IRS for each case was assigned as the staining result for the patient. The specimens were rescored if the difference between the scores determined by the two pathologists was >3.

### Cell culture and RNA interference

The human NPC cell lines CNE1 and CNE2 were maintained in RPMI-1640 supplemented with 10% fetal bovine serum (Invitrogen, Carlsbad, CA) and 1% penicillin-streptomycin, and were cultured at 37°C in a humidified atmosphere of 5% CO2. siRNA against ULK1 (Santa Cruz Biotechnology) was transfected into NPC cells in six-well plates using Lipofectamine 2000 (Invitrogen) according to the manufacturer’s instructions.

### MTT assay and apoptosis detection

Cell viability was measured by a 3-(4, 5-dimethylthiazol-2-yl)-2, 5-diphenyl tetrazolium bromide (MTT) assay (Sigma, St, Louis, Missouri, USA). The OD value was measured at 550 nm with a 655 nm reference filter by microplate reader (Bio Rad, Hercules, CA).

The apoptosis assay was conducted by flow cytometry using Annexin V–fluorescein isothiocyanate (FITC) and propidium iodide (PI) stains according to manufacturers' instructions (BioVision Inc). Each sample was then subjected to analyses by flow cytometry (Beckman Coulter, cytomics FC 500, CA).

### Western Blotting Analysis

Equal amounts of NPC tissue lysates were resolved by SDS-polyacrylamide gel electrophoresis (PAGE) and electrotransferred on a polyvinylidene difluoride (PVDF) membrane (Pall Corp., Port Washington, NY). The tissues were then incubated with primary rabbit monoclonal antibodies against ULK1 (Abcam, #ab65056, Cambridge, UK). The immunoreactive signals were detected with enhanced chemiluminescence kit (Amersham Biosciences, Uppsala, Sweden). The procedures followed were conducted in accordance with the manufacturer’s instructions.

### Selection of Cutoff Score

X-tile plots were created for assessment of ULK1 expression and optimization of cutpoints based on outcome[28]. The X-tile program divided the cohorts randomly into a matched training and validation set as a method for selecting optimal cutpoints, respectively. Statistical significance was assessed by using the cutoff score derived from a training set to parse a separate validation set, using a standard log-rand method, with P values obtained from a lookup table. The X-tile plots allowed determination of an optimal cutoff value while correcting for the use of minimum P statistics by Miller-Siegmund P-value correction.

### Statistical analysis

Statistical analysis was performed with SPSS software (SPSS Standard version 13.0, SPSS, Chicago, IL, USA). The chi-square test or Fisher’s exact test was employed to evaluate the relationship between ULK1 expression and clinicopathological variables. Pearson’s chi-squared test was used to analyze the relationship between ULK1 expression and therapeutic response. Survival curves were plotted by the Kaplan–Meier method and compared by the log-rank test. Multivariate survival analysis was performed on all parameters that were found to be significant on univariate analysis using the Cox regression model. P-values of < 0.05 were considered significant.

## Result

### Patients’ Characteristics

The clinicopathologic characteristics of the NPC patients are presented in Table 1. In our training cohort, a total of 141 patients were treated by RT only, where 101 patients were treated with IC plus RT. A total of 93 patients received IC prior to concurrent RCT. At the evaluation time, CR, PR, NC and PD were achieved in 258, 30, 29, and 8 patients, respectively. In the validation cohort, 87 patients received radiotherapy only, where 66 patients were treated with IC plus RT. In addition, 62 patients received IC prior to concurrent RCT. At the evaluation time, CR, PR, NC and PD were achieved in 164, 20, 20 and 11 patients, respectively.

**Table 1:** The association of ULK1 expression with clinicopathological variables.

	Training cohort				Validation cohort			
Variables	Case	low expression	high expression	P value^b^	Case	low expression	high expression	P value^b^
Age		146	189			96	119	
⩽57^a^	159	66	93		109	50	59	
>57	176	80	96	0.467	106	46	60	0.715
Gender								
Male	273	116	157		152	69	83	
Female	62	30	32	0.398	63	27	36	0.733
WHO type								
Type III	259	117	142		188	88	100	
Type II	76	29	47	0.228	27	8	19	0.093
T status								
T1–2	140	72	68		80	40	40	
T3–4	195	74	121	0.014*	135	56	79	0.225
N status								
N0–1	191	89	102		126	62	64	
N2–3	144	57	87	0.200	89	34	55	0.110
Clinical stage								
I+II	79	44	35		38	23	15	
III+IV	256	102	154	0.013*	176	72	104	0.034*
RT response								
CR	122	59	63		66	34	32	
Non-CR	19	4	15	0.026*	21	5	16	0.026*
IC/RT response								
CR	72	42	30		51	28	23	
Non-CR	29	8	21	0.005*	15	3	12	0.017*
IC/RCT response								
CR	64	28	36		47	25	22	
Non-CR	29	5	24	0.013*	15	1	14	0.001*

a Mean age

b Chi-square test

RT, radiotherapy; CR, complete response; Non-CR (including PR, partial response; NC, no change; PD, progressive disease); IC, induction chemotherapy; RCT, radiochemotherapy

*Statistically significant difference

### Up-regulation of ULK1 in NPC tissues

**Fig 1:**
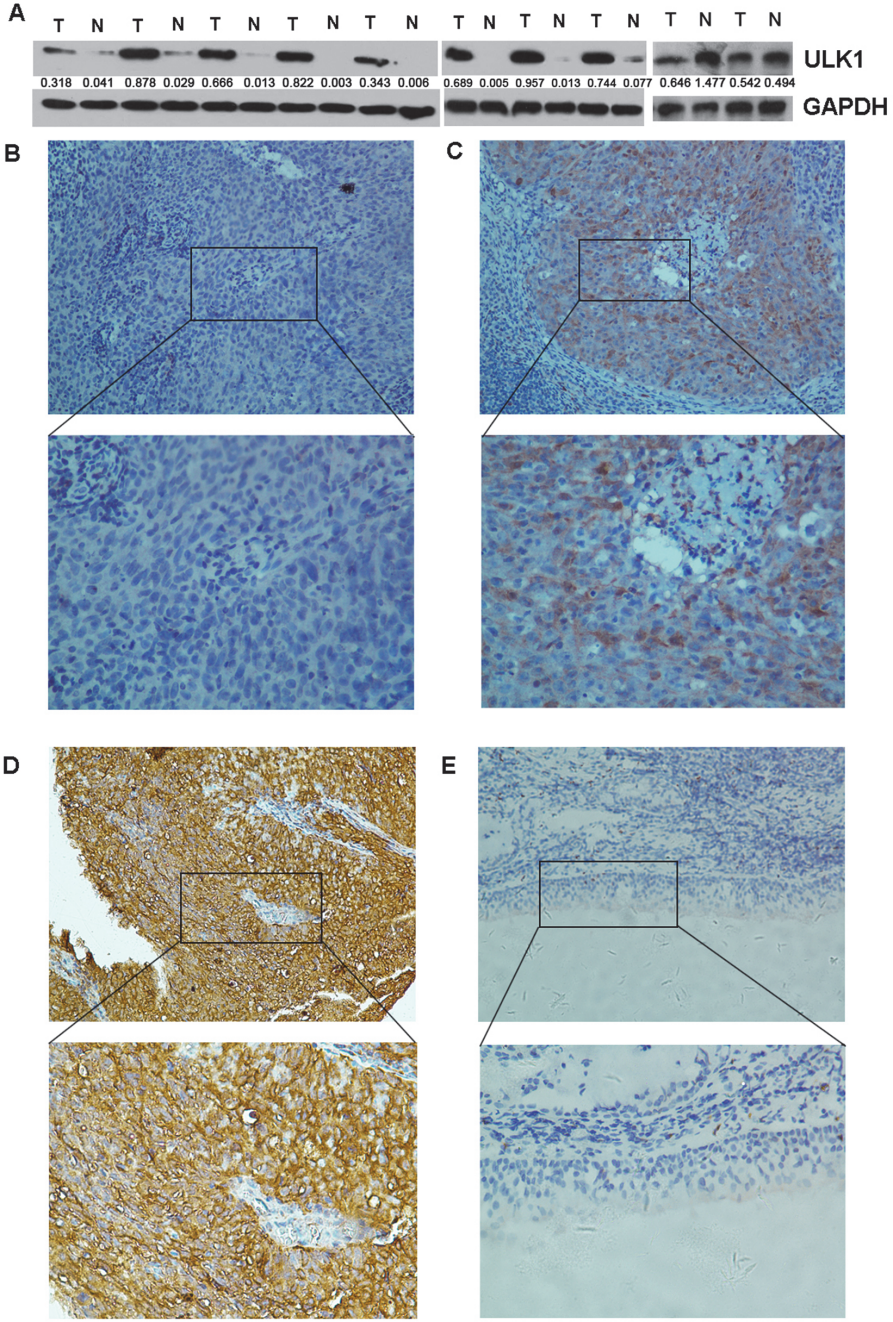
The expression of ULK1 in NPC tissues. (A) Western blot analysis of ULK1 protein expression in 10 representative paired of NPC (T) and adjacent normal mucosa tissues (N). Equal loading of protein was determined by GAPDH. The number below indicated the expression level of ULK1 relative to GAPDH in each samples. (B) An NPC case demonstrated low expression of ULK1, in which negative immunohistochemistry staining was observed in all the NPC cells (upper panel ×200). (C) An NPC case showed moderate ULK1 staining (upper panel ×200). (D) Strong ULK1 IHC signaling was detected in the cytoplasm pattern of the NPC cancer cells (upper panel ×200). (E) Normal nasopharyngeal mucosa tissue showed negative expression of ULK1 protein (upper panel ×200). The lower panels indicated the higher magnification (× 400) from the area of the box in B., C., D. and E., respectively.

The expression of ULK1 was examined in 10 paired of NPC and ANTs. Our western blotting analysis demonstrated that the 8 out of 10 (80.0%) NPC cases also exhibited up-regulated ULK1 expression in protein level as compared to that in ANTs (Fig. 1A). For IHC staining, the positive ULK1 protein immunoreactivity was mainly detected in the cytoplasm pattern of tumor cells (Fig. 1B-D). Immunoreactivity score of ULK1 protein ranged from 0 to 12. On the other hand, among the 50 non-malignant tissues, absent or weak immunoreactivity staining of ULK1 protein was detected (Fig. 1E). In addition, we collected pairs of distant metastasis as well as pairs of recurrent NPC to compare their expression level in comparison with the primary NPC respectively. As shown in S1 Fig., in both distant metastasis and recurrent NPC, the expression level of ULK1 was much higher (P<0.05).

### Selection of Cutoff Score for High Expression of ULK1

**Fig 2:**
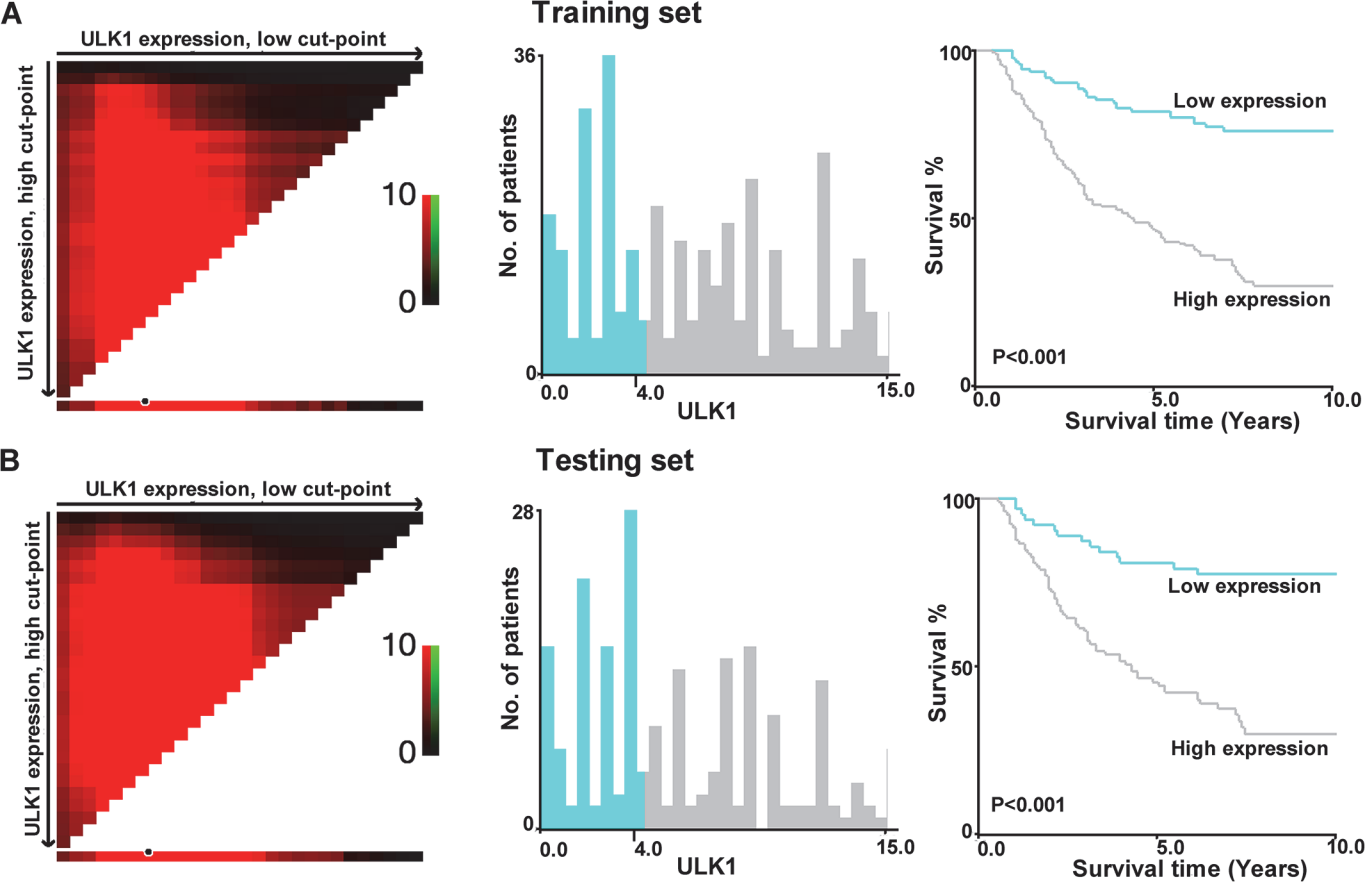
X-title plots of ULK1 expression for optimal cut-point selection in the training cohort. Patients in the training cohort were randomly divided into equal training (A) and pretesting subsets (B). The cut-point for the training set (IHC score > 4, P<0.001) is demarcated by the circle (black with white border), which was then used as the point separating low ULK1 expression (blue) from high ULK1 expression (gray) in the expression frequency of the whole set (middle panel). The right panel presents the Kaplan-Meier curve for examining the survival of cohort subsets defined by ULK1 expression ⩽4(blue line) and >4 (gray line).

To develop a reasonable cutoff score and avoid the problems of multiple cutpoint selection, the X-tile program was employed to determine cutoff score for ULK1 expression. According to the X-tile plots, we dichotomized the training cohort into low (IHC score ≤4) and high (IHC score >4) expression subgroups based on a cut-point of more than IHC score 4 for high ULK1 expression, which achieved high statistical significance (Fig. 2). In the training cohort, high expression of ULK1 was observed in 189/335 (56.4%) of NPC samples, and in 7/45 (15.6%) normal nasopharyngeal mucosal tissues (P<0.001); in the validation cohort, high expression of ULK1 was observed in 119/215 (55.3%) of NPC samples according this generated cut-off point.

### Correlation of ULK1 expression with clinicopathologic variables

Table 1 summarized the detailed information about the rates of high ULK1 expression with respect to several standard clinicopathological features in these two cohorts. A statistically significant correlation between ULK1 expression with T status, and overall clinical stage was observed in training cohort (P<0.05). In validation cohort, high ULK1 expression was observed to be associated with overall clinical stage (P<0.05). However, we failed to detect a relationship between ULK1 expression and other patient characteristics, including patient's age, gender, and WHO pathological type (P > 0.05).

### Correlation between ULK1 expression and therapeutic response

In the training and validation cohort, primary CR was achieved in 259/335 (77.3%) and 161/215 (74.9%) of the NPC patients, respectively. And the remaining cases were included in the Non-CR group (PR/NC/CD). Moreover, using the optimal cut-off value of more than 4 score, ULK1 expression was also the factor that showed a negative correlation with treatment response in the RT, IC/RT as well as IC/RCT groups both in the training and validation cohort, in which high expression of ULK1 was observed more frequently in Non-CR subset than in CR subset (P<0.05, Table 1).

**Fig 3:**
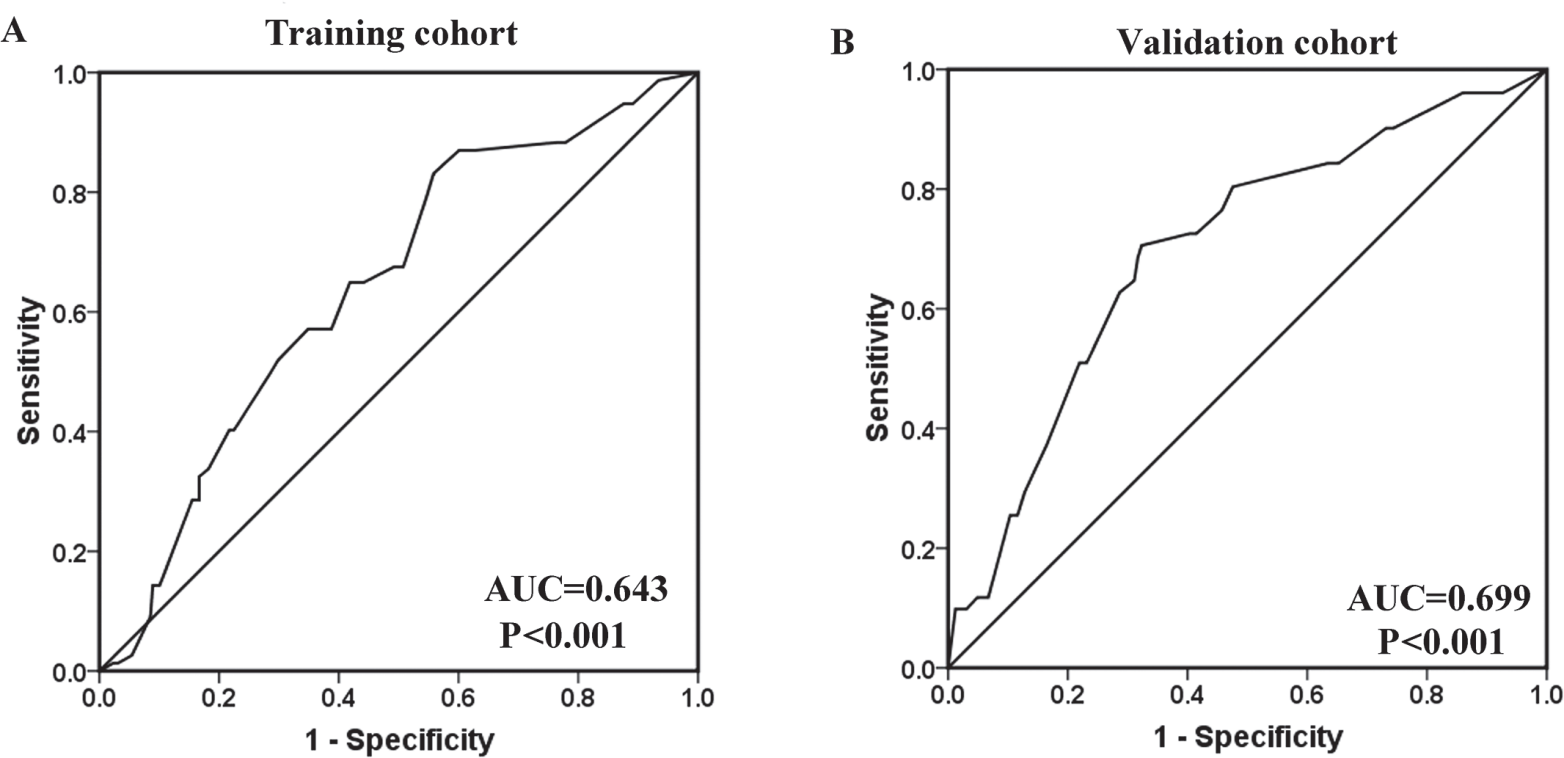
The predictive value of ULK1 expression regarding NPC patients’ treatment response. Receiver operating characteristic curve analysis for ULK1 expression was performed to assess NPC treatment response in the training cohort (AUC = 0.643, P<0.001) (A) and in the validation cohort (AUC = 0.699, P<0.001) (B).

We than applied the ROC curve analysis method to explore the predictive value of expression in therapeutic response in training and validation cohort. For ROC curve analysis, the treatment response was dichotomized: CR versus PR+NC+PD. The result showed a promising predictive value of regarding to treatment response in both training cohorts (AUC = 0.643, P<0.001) and validation cohort (AUC = 0.699, P<0.001) (Fig. 3).

### Association between Clinicopathologic Features, ULK1 Expression, and Patient Survival: Univariate Survival Analysis

**Fig 4:**
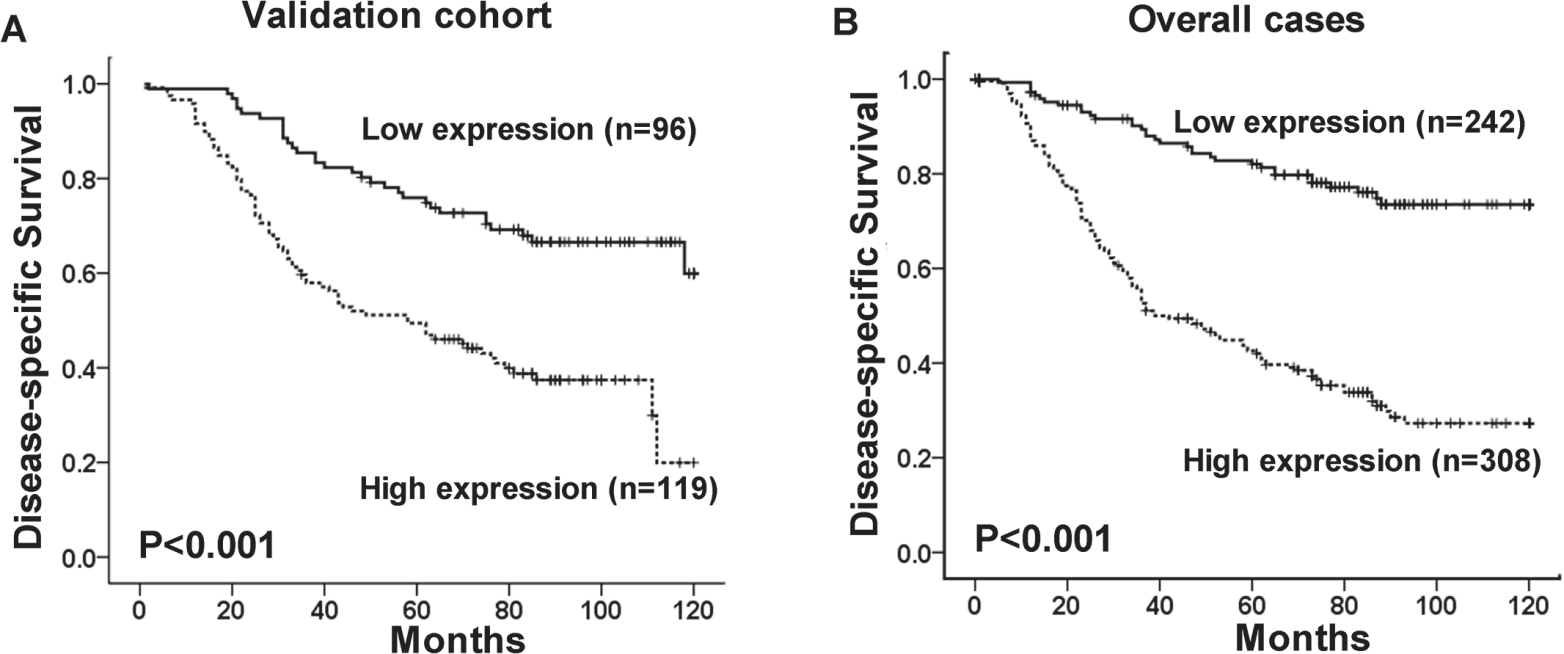
The association between ULK1 expression and NPC patients’ survival. Kaplan-Meier survival analysis of ULK1 expression for disease-specific survival in the validation cohort (A) and in the overall cases (B) (log-rank test).

As shown in Fig. 4, Kaplan-Meier analysis showed that the DSS difference between subsets with high and low ULK1 expression was marginal in the validation and overall patients (P<0.001). To confirm the representativeness of the NPCs, we first tested well-established prognostic factors of patient survival. In the validation cohort, univariate analysis evaluated a significant impact of well-known clinical pathological prognostic parameters on patients’ survival, such as T status, overall clinical stage, as well as therapeutic response (P<0.05, Table 2). Assessment of patient survival also revealed that high expression of ULK1 was significantly correlated with poor DSS in the validation cohort (HR = 2.697, 95%CI (1.770–4.018); P<0.001, Table 2).

**Table 2:** Univariate analysis of ULK1 expression and various clinicopathological parameters in validation and overall cases of NPC patients.

	Validation cohort				Overall cases			
Variables	Case	HR	95% CI	*P*-value	Case	HR	95% CI	*P*-value
Age								
⩽57^a^	109	1.0			268	1.0		
>57	106	1.445	0.983–2.124	0.061	282	1.351	0.993–1.842	0.055
Gender								
Female	152	1.0			425	1.0		
Male	63	1.474	0.941–2.308	0.090	125	1.163	0.960–1.410	0.123
WHO grade								
Type III	188	1.0			447	1.0		
Type II	27	1.202	0.685–2.109	0.522	103	1.073	0.746–1.544	0.703
T status								
T1–2	80	1.0			220	1.0		
T3–4	135	1.693	1.114–2.571	0.014*	330	1.598	1.156–2.209	0.005*
N status								
N0–1	126	1.0			317	1.0		
N2–3	89	1.34	0.914–1.963	0.134	233	1.612	1.184–2.195	0.002*
Clinical stage								
I+II	38	1.0			117	1.0		
III+IV	176	2.779	1.446–5.343	0.002*	432	2.658	1.679–4.207	<0.001*
Therapeutic response								
CR	164	1.0			422	1.0		
Non-CR	51	4.395	2.951–6.546	<0.001*	128	2.378	1.716–3.294	<0.001*
ULK1 expression								
Low expression	96	1.0			242	1.0		
High expression	119	2.697	1.770–4.108	<0.001*	308	4.206	2.875–6.153	<0.001*

a Mean age.

Cox propotional hazard regression model, enter; HR, Hazard ratio; CI, confidence interval; CR, complete response, Non-CR (including PR, partial response; NC, no change; PD, progressive disease)

*Statistically significant difference

Results in the overall cases were similar to those in the validation cohort. Patients with high ULK1 expression also displayed a significant trend toward worse survival compared with patients with low ULK1 expression (HR = 4.206, 95%CI (2.875–6.153) P<0.001, Table 2). Of the other prognostic factors, univariate analysis demonstrated that T stage, N stage, overall clinical stage, well as therapy response were also significant predictors for patients’ DSS (P<0.05, Table 2).

### Independent Prognostic Factors of NPC: Multivariate Survival Analysis

Since variables observed to have prognostic influence by univariate analysis might covariate, the expression of ULK1 as well as other clinicopathologic features that were significant in univariate analysis was examined in multivariate analysis. We found that high expression of was evaluated as an independent risk factor for adverse DSS in validation cohort (hazard ratio, 3.653; 95% confidence interval [CI], 1.996; 95% CI, 1.286–3.097, P = 0.002; Table 3). The same results were obtained in our overall cases (hazard ratio, 3.609; 95% CI, 2.442–5.332, P<0.002; Table 3). Of the other variables, CRT response and overall clinical stage were found as independent prognostic factors for patient survival in both validation cohort and overall patients (P<0.05).

**Table 3:** Results of multivariate Cox proportional-hazards analysis for disease-specific survival for NPC patients.

Variable	HR	95% CI	*P*-value
**Validation cohort**			
Tumor status (T1+2 vs. T3+4)	1.088	0.675–1.7563	0.729
Clinical stage (I+II vs. III+IV)	2.198	1.036–4.664	0.040
Therapeutic response (CR vs. Non-CR)	3.493	2.307–5.287	<0.001*
ULK1 expression (Low vs. High)	1.996	1.286–3.097	0.002*
**Overall cases**			
T status (T1+2 vs. T3+4)	1.008	0.669–1.521	0.968
Nodel status (N0+1 vs. N2+3)	1.153	0.791–1.682	0.459
Clinical stage (I+II vs. III+IV)	1.904	1.021–3.551	0.043*
Therapeutic response (CR vs. Non-CR)	1.509	1.068–2.133	0.020*
ULK1 expression (Low vs. High)	3.609	2.442–5.332	<0.001*

Cox propotional hazard regression model, enter; HR, Hazard ratio; CI, confidence interval; CR, complete response, Non-CR (including PR, partial response; NC, no change; PD, progressive disease)

*Statistically significant difference

### Silencing of ULK1 inhibits cellular growth and promotes apoptosis in NPC cells

To exam biological role of ULK1 in NPC, we used ULK1 genes-specific siRNA in CNE1 and CNE2 cell lines, which effectively knocked down the expression of endogenous ULK1 protein (S2A Fig.). A MTT assay revealed that knock down of ULK1 could cause a significant reduction of cellular growth in cell lines (S2B Fig.). Furthermore, we used flow cytometry analysis to detect that the percentage of apoptosis cells were significantly increased in both the cell lines. These results suggested that suppression of pro-oncogenic role of ULK1 in NPC cell lines (S2C Fig.).

### New prognostic model

**Fig 5:**
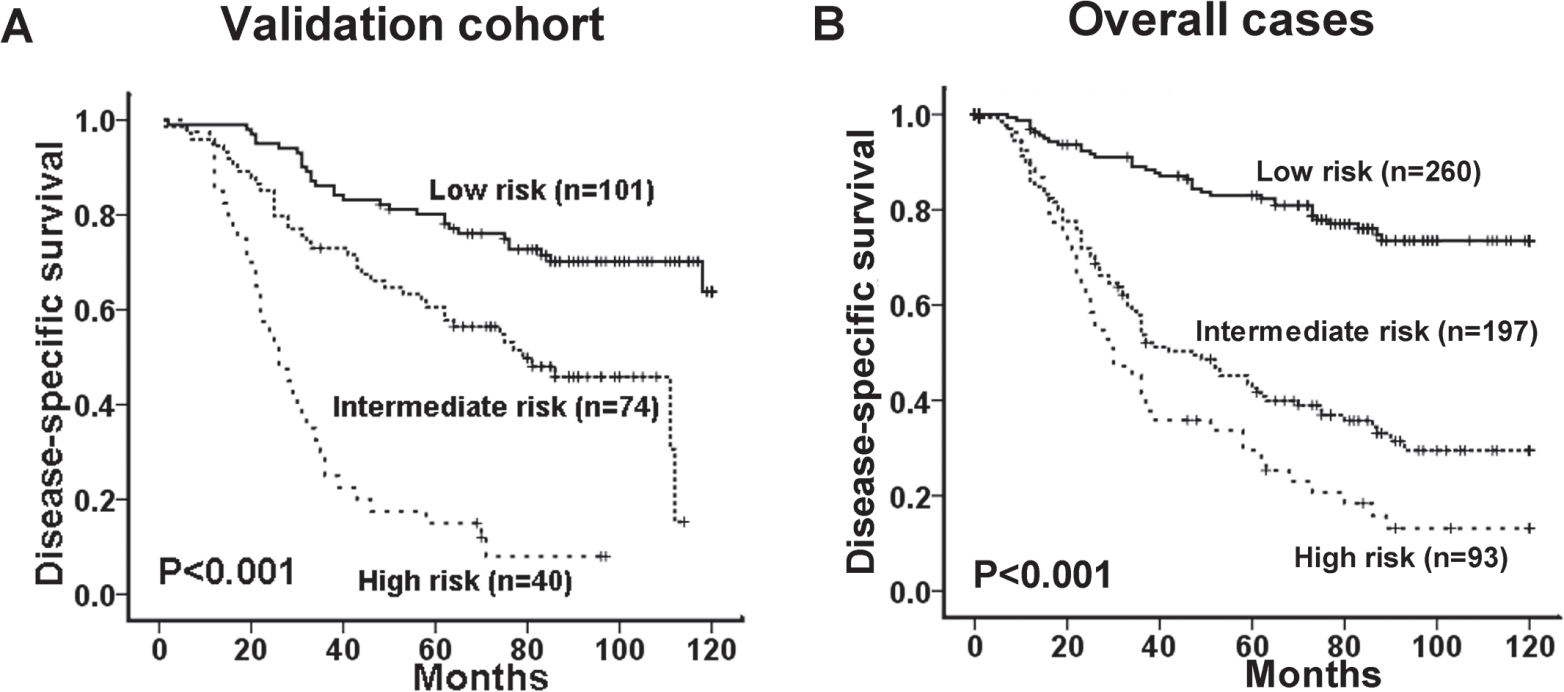
Comparison of disease-specific survival based on a novel clinico-pathological prognostic model (including ULK1 expression, therapy response and overall clinical stage) in the validation cohort (A) and overall cases (B) (log-rank test).

Based on the results of multivariate survival analysis, we proposed a new clinico-pathological prognostic model composed of three variables: high ULK1 expression, advanced clinical stage and poor CRT response. Patients were categorized into three risk group: low-risk (0 risk factor), intermediate-risk (1 risk factor), and high risk (2–3 risk factors) groups. On Kaplan-Meier analysis, the 3 risk groups showed clear separation into 3 survival groups in the validation cohort (P<0.001, the specificity and sensitivity were 65.1% and 71.7%, Fig. 5A). Patients in the low- (n = 101), intermediate- (n = 74), and high- (n = 40) risk groups had a 5-year DSS of 80%, 61% and 15% respectively. For the overall cases, the new prognostic model also could differentiate different patients with different outcomes (5-year DSS of low- (n = 260), intermediate- (n = 197), and high- (n = 93) risk group was 83%, 45%, and 30% respectively) (P<0.001, the specificity and sensitivity were 70.1% and 77.2%, Fig. 5B).

## Discussion

Previous studies have shown autophagy-related proteins are associated with patients prognosis in several human cancers. ULK1, a key protein in autophagy, has been suggested the pro-oncogeneic role in human ESCC and breast cancer[24,25]. To best of our knowledge, the prognostic value of ULK1 in NPC has never been studied. Thus, in the present study, we selected the NPC specimens from two independent cohorts to detect their ULK1 protein expression pattern and survival probability. Of note, to avoid predetermined arbitrary cutpoint, we applied X-tile plots to obtain the optimal IHC score for high UKL1 expression. For validation, this X-tile-derived cutoff point was subjected to analysis of the association of ULK1 expression with patient outcome and clinical characteristics in validation cohort and overall cases.

The role of autophagy with the context of cancer remains somewhat controversial and appears to be quite divergent in the pre- and postmalignant states[29,30]. Authophagy has been well established to play an important role in the development and progression of cancer. However, whether antuophagy in cancer cells results in death or cell-protection remains controversial[12,31]. For example, high expression of Beclin 1, one of the first identified mammalian autophagy proteins, has been observed to be associated with either favourable or inferior prognosis in different types of human cancers[32,33,34]. As a key autophagosomal modulating protein, ULK1 was generally identified as an oncogene and has been detected to be up-regulated in human ESCC and breast cancer[24,25]. Consistent with the previous result, our study also revealed elevated ULK1 expression in NPC tissues. In addition, further correlation analysis demonstrated that high expression of ULK1 in NPC was positively correlated with tumor aggressive and advanced clinical stage. These data, taken together, suggested that up-regulation of a major autophagy component, ULK1 might facilitate the progressive process of human maligancies, including NPC.

We know that the maliganent cells always have a high demand for nutrient and oxygen to facilitate their high proliferation rate during tumor development and progression. However, during the long term of therapy courses, the NPC tumor cells often encounter unfavourable condition, such as metabolic stress and severe hypoxia due to the decreased microvascular density (MVD) [35,36]. The function of autophagy to provide energy, maintain cellular hemeostasis and degrade toxic cytoplasmic constituents helps to keep cancer cells alive during nutrient and oxygen deprivation and other stressfull condition. Therefore, induction of autophagy has emerged as a drug resistance mechanism that promotes cancer cell survival and evades hypoxic cell death via self-digestion. Accordingly, NPC cells with elevated ULK1 expression, interpreted as presenting an autophagy phenomenon, possess the potential to activate initiation of autophagy and protect them form both apoptosis and necrosis. This might provide a better understanding of why NPC patents have a high expression of ULK1 was closely linked with poor therapeutic response. In this respect, ULK1 had been suggested as a promising therapeutic molecular target for NPC diseasing control. Furthermore, it is noteworthy that acquisition of chemo/radio-resistance under therapy may affect the biology of tumor cell, leading toward a more malignant phenotype in cancer progression. This is could explain the phenomenon that a higher frequency of elevated ULK1 protein was observed in recurrent and distant metastasis NPCs which were acquired with a more aggressive phenotype and worse survival outcome. Our finding, combined together, suggested that high ULK1 expression may favor the NPC cells a more aggressive phenotype, surviving form chemo/radiotherapy and metastastsizing to distant organs, suggesting a potential molecular target for NPC therapy. Of note, more detailed investigation are required to the development of a novel strategy for the adjuvant therapy of NPC.

As for the prognostic impact of ULK1 on NPC, our study demonstrated that for the first time high ULK1 expression was closely correlated with adverse survival for NPC patients. This result is closely consistent with the previous studies, which have reported that elevated ULK1 expression is associated with poor survival in ESCC and breast cancer patients[24,25]. IHC is perhaps the most readily adaptable method to clinical practice, as it is already widely used to guide treatment of patients[24,25]. Recently, IHC staining of ULK1 was validated for the detection of antophagy in several different human paraffin-embedded tumors. Thus, ULK1 is an attractive biomarker for autophagy that could be used in many diseases, especially cancer. Our reports suggested that the examination of ULK1 expression, as detected by IHC method, could be used as an effective additional tool to predict the therapeutic response and prognostic outcome of NPC and make the optimal clinical decisions. For example, those high-risk patients with high ULK1 expression could be offered for higher-dose radiation and adjuvant chemotherapy earlier at the course of therapy modality. By contrast, low-risk NPC patients with lower ULK1 expression, may benefit from mild treatment without unnecessary radical therapy.

Our study also suffered from some limitations. As we know, ULK1 kinase activity affects its autophary functions, however we only analyzed the total ULK1 expression level in NPC tissues in the present study. If phosphorylated levels of ULK1 in therapeutic response or resistant NPC tissues could be examined, the result could be more convincing.

Our study demonstrated that ULK1 was an independent prognostic biomarker for therapeutic response in NPC, and examination of ULK1 expression by IHC method might be helpful in determining the prognosis of this tumor in clinical practice. Furthermore, inhibitor of ULK1 could be emerged as a novel promising cancer therapeutic strategy by compromising autophagic cell survival.

## Supporting Information

S1 Fig ULK1 expression is upregulated in recurrent (A) and distant metastasis (B) NPC samples. Left panel: IHC staining of representative of ULK1 expression in recurrent/distant metastasis NPC with the paired primary NPC sample. Right panel: statistical analysis revealed that a significant increase of ULK1 expression in recurrent/distant metastasis NPC relative to expression in primary NPC.
(JPG)

S2 Fig Knockdown of endogenous ULK1 inhibited NPC cellular growth and promoted apoptosis. (A) Western blotting analysis for ULK1 expression in NPC cells transfected with ULK1 or control siRNA, respectively. (B) Silencing of ULK1 inhibited cellular growth as determined by MTT assay. Each bar represented the average + SD of three independent experiments. (C) Silencing of ULK1 promoted more apoptosis in NPC cells as determined by flow cytometry analysis.
(JPG)
