# Supplementary figures and images for: ULK1: A Promising Biomarker in Predicting Poor Prognosis and Therapeutic Response in Human Nasopharygeal Carcinoma
Source: PLoS One. 2015 Feb 25;10(2):e0117375. doi: 10.1371/journal.pone.0117375 (PMC4340914; doi:10.1371/journal.pone.0117375)

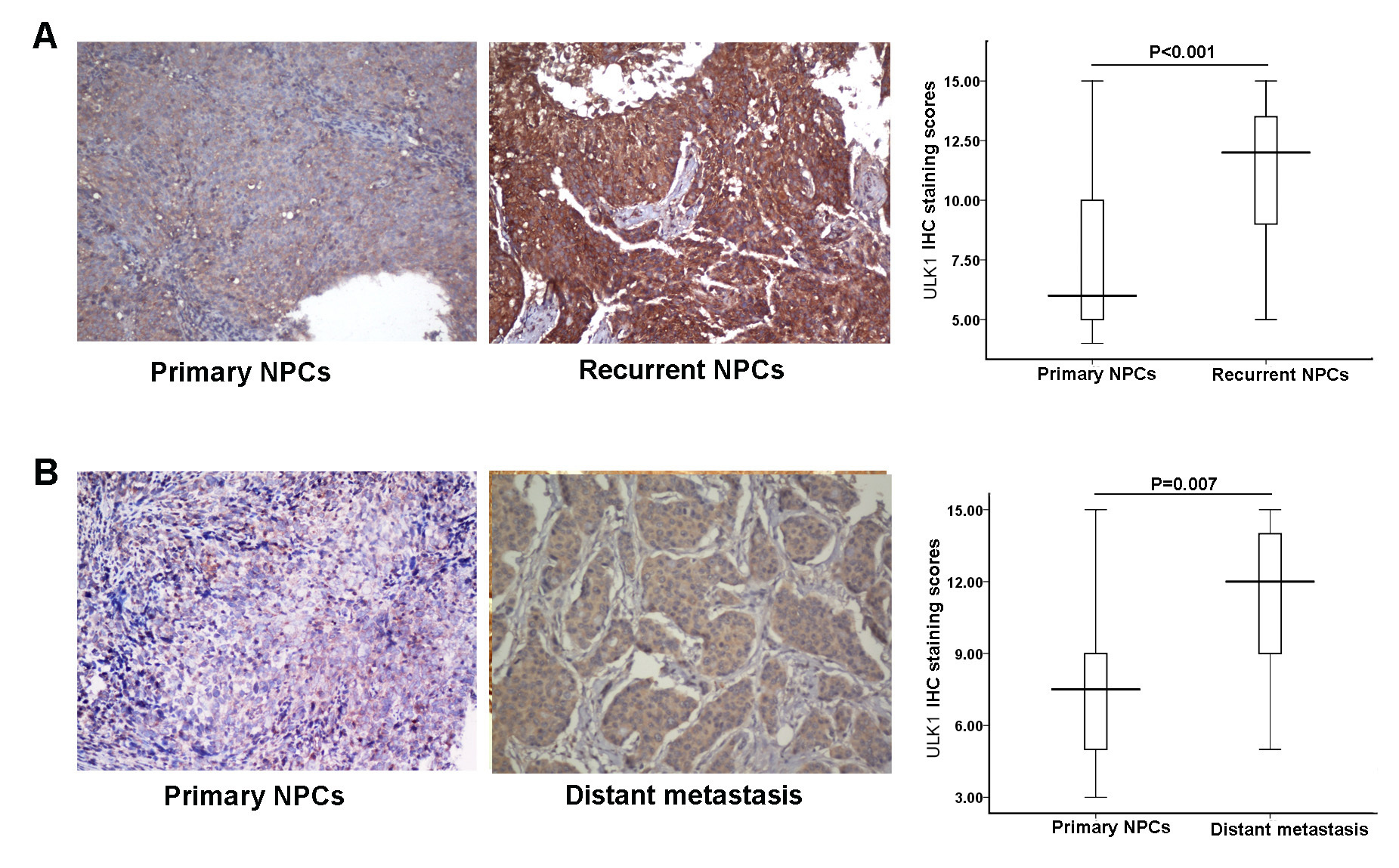

Supplement: S1 Fig — Left panel: IHC staining of representative of ULK1 expression in recurrent/distant metastasis NPC with the paired primary NPC sample. Right panel: statistical analysis revealed that a significant increase of ULK1 expression in recurrent/distant metastasis NPC relative to expression in primary NPC. (JPG) [file pone.0117375.s001.jpg]

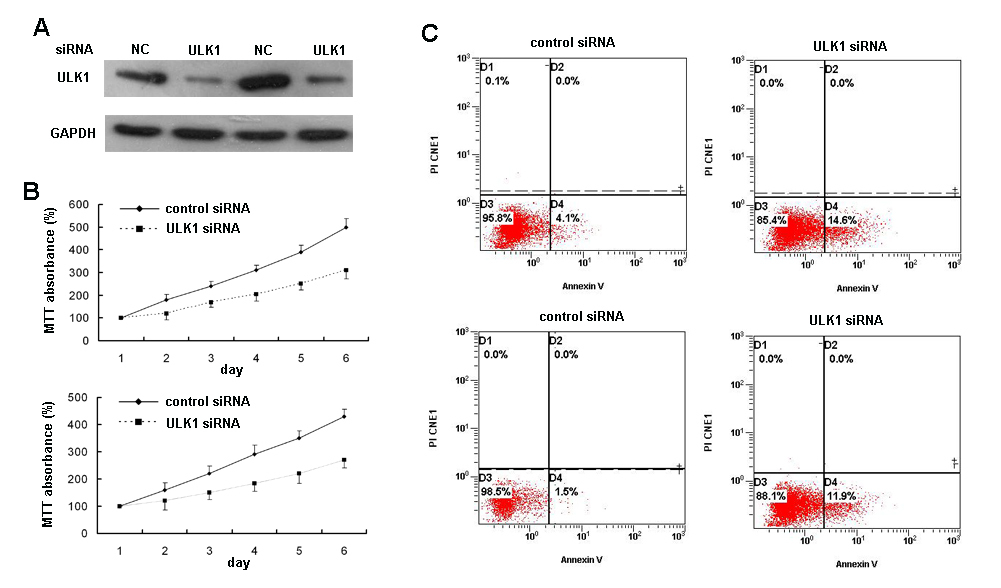

Supplement: S2 Fig — (A) Western blotting analysis for ULK1 expression in NPC cells transfected with ULK1 or control siRNA, respectively. (B) Silencing of ULK1 inhibited cellular growth as determined by MTT assay. Each bar represented the average + SD of three independent experiments. (C) Silencing of ULK1 promoted more apoptosis in NPC cells as determined by flow cytometry analysis. (JPG) [file pone.0117375.s002.jpg]
